# Cediranib combined with carboplatin and paclitaxel in patients with metastatic or recurrent cervical cancer (CIRCCa): a randomised, double-blind, placebo-controlled phase 2 trial

**DOI:** 10.1016/S1470-2045(15)00220-X

**Published:** 2015-11

**Authors:** R Paul Symonds, Charlie Gourley, Susan Davidson, Karen Carty, Elaine McCartney, Debbie Rai, Susana Banerjee, David Jackson, Rosemary Lord, Mary McCormack, Emma Hudson, Nicholas Reed, Maxine Flubacher, Petra Jankowska, Melanie Powell, Caroline Dive, Catharine M L West, James Paul

**Affiliations:** a Department of Cancer Studies, University of Leicester, Leicester, UK; b University of Edinburgh Cancer Research UK Centre, MRC IGMM, Edinburgh, UK; c The Christie NHS Foundation Trust, Manchester, UK; d Cancer Research UK Clinical Trials Unit, Institute of Cancer Sciences, University of Glasgow, Glasgow, UK; e Gynaecology Unit, Royal Marsden Hospital NHS Foundation Trust, London, UK; f Institute of Oncology, Leeds Cancer Centre, St James's University Hospital, Leeds UK; g Clatterbridge Centre for Oncology, University of Liverpool, Liverpool, UK; h Oncology Department, University College Hospital, London, UK; i Velindre Hospital, Cardiff, UK; j Beatson West of Scotland Cancer Centre, Gartnavel General Hospital, Glasgow, UK; k Oncology Department, Poole Hospital, Dorset, UK; l Musgrove Park Hospital, Taunton and Somerset NHS Foundation Trust, Taunton, UK; m St Bartholomew's Hospital, London, UK; n Cancer Research UK Manchester Institute, University of Manchester, Manchester, UK; o Institute of Cancer Sciences, University of Manchester, Manchester, UK

## Abstract

**Background:**

Patients treated with standard chemotherapy for metastatic or relapsed cervical cancer respond poorly to conventional chemotherapy (response achieved in 20–30% of patients) with an overall survival of less than 1 year. High tumour angiogenesis and high concentrations of intratumoural VEGF are adverse prognostic features. Cediranib is a potent tyrosine kinase inhibitor of VEGFR1, 2, and 3. In this trial, we aimed to assess the effect of the addition of cediranib to carboplatin and paclitaxel chemotherapy in patients with metastatic or recurrent cervical cancer.

**Methods:**

In this randomised, double-blind, placebo-controlled phase 2 trial, which was done in 17 UK cancer treatment centres, patients aged 18 years or older initially diagnosed with metastatic carcinoma or who subsequently developed metastatic disease or local pelvic recurrence after radical treatment that was not amenable to exenterative surgery were recruited. Eligible patients received carboplatin AUC of 5 plus paclitaxel 175 mg/m^2^ by infusion every 3 weeks for a maximum of six cycles and were randomised centrally (1:1) through a minimisation approach to receive cediranib 20 mg or placebo orally once daily until disease progression. The stratification factors were disease site, disease-free survival after primary therapy or primary stage IVb disease, number of lines of previous treatment, Eastern Cooperative Oncology Group performance status, and investigational site. All patients, investigators, and trial personnel were masked to study drug allocation. The primary endpoint was progression-free survival. Efficacy analysis was by intention to treat, and the safety analysis included all patients who received at least one dose of study drug. This trial is registered with the ISCRTN registry, number ISRCTN23516549, and has been completed.

**Findings:**

Between Aug 19, 2010, and July 27, 2012, 69 patients were enrolled and randomly assigned to cediranib (n=34) or placebo (n=35). After a median follow-up of 24·2 months (IQR 21·9–29·5), progression-free survival was longer in the cediranib group (median 8·1 months [80% CI 7·4–8·8]) than in the placebo group (6·7 months [6·2–7·2]), with a hazard ratio (HR) of 0·58 (80% CI 0·40–0·85; one-sided p=0·032). Grade 3 or worse adverse events that occurred in the concurrent chemotherapy and trial drug period in more than 10% of patients were diarrhoea (five [16%] of 32 patients in the cediranib group *vs* one [3%] of 35 patients in the placebo group), fatigue (four [13%] *vs* two [6%]), leucopenia (five [16%] *vs* three [9%]), neutropenia (10 [31%] *vs* four [11%]), and febrile neutropenia (five [16%] *vs* none). The incidence of grade 2–3 hypertension was higher in the cediranib group than in the control group (11 [34%] *vs* four [11%]). Serious adverse events occurred in 18 patients in the placebo group and 19 patients in the cediranib group.

**Interpretation:**

Cediranib has significant efficacy when added to carboplatin and paclitaxel in the treatment of metastatic or recurrent cervical cancer. This finding was accompanied by an increase in toxic effects (mainly diarrhoea, hypertension, and febrile neutropenia).

**Funding:**

Cancer Research UK and AstraZeneca.

## Introduction

Although the incidence of cervical cancer has decreased in the UK since 1988,^1^ it remains the fourth most common cancer in women worldwide.^2^ Radical surgery and chemoradiotherapy are associated with high cure rates, but treatment options for patients who develop metastatic disease or relapse within the irradiated pelvis are very unsatisfactory.

A Gynecologic Oncology Group study^3^ comparing four cisplatin-containing doublet combination chemotherapy regimens used to treat patients with stage IVb recurrent or persistent cervical carcinoma reported typical results for patients with advanced cervical cancer. The proportion of patients with a response in the four groups of the study ranged from 22·3% to 29·1%, and the median overall survival was between 9·99 months and 12·87 months. The most effective combination seemed to be cisplatin plus paclitaxel, although the difference in overall survival between the four treatment groups was not statistically significant.

**Research in context**

**Evidence before this study**

We searched PubMed between January, 1990, and January, 2010, using the terms “cervical cancer”, “chemotherapy”, “advanced”, “metastatic”, ”recurrent”, and “trial”. Articles published in languages other than English were excluded. From phase 3 results, the combination of carboplatin and paclitaxel was taken as the standard of care. The addition of “VEGF” to the search term found no trials targeting VEGF in cervical cancer published before the start of the study.

**Added value of this study**

The results of our phase 2 study provide evidence that cediranib might have therapeutic activity in recurrent or metastatic cervical cancer when added to carboplatin and paclitaxel.

**Implications of all the available evidence**

A phase 3 trial reported during the follow-up of patients in this trial showed that the addition of bevacizumab to combination chemotherapy in patients with advanced cervical cancer improved median overall survival. Taken together with the results from the CIRCCa study, further study of VEGF inhibition is warranted in this disease setting.

High tumour angiogenesis is associated with poor survival when cervical cancer is treated with radiotherapy,^4^ and high tumour vascularity is a notable prognostic factor that is independent of intrinsic tumour radiosensitivity.^5^ Loncaster and colleagues^6^ reported a significant association of VEGF expression in cervical cancer biopsies with overall survival (p=0·0008) and metastasis-free survival (p=0·0062), but not with local control (p=0·23), in a group of 100 patients with bulky stage Ib–IIIb tumours treated by radical radiotherapy. A review of several tissue marker studies in cervical cancer emphasised the importance of increased tumour VEGF as an adverse prognostic factor.^7^ Cediranib is a potent inhibitor of the tyrosine kinase activity of VEGFR1, 2, and 3, and c-KIT. The drug disrupts VEGF signalling pathways in endothelial and cancer cells and was therefore believed to be a potentially effective agent that could be added to conventional chemotherapy.^8^

The rationale for this trial was the notion that no satisfactory treatment is available for recurrent or metastatic cervical cancer, although platinum-based combinations are regarded as the present standard of care. In the selection of a chemotherapeutic regimen, toxicity needs to be balanced against efficacy. Paclitaxel plus carboplatin is a widely used treatment for relapsed cervical cancer, partly owing to familiarity as a result of its use in ovarian cancer and partly because of the assumption that it is likely to have similar activity with less toxicity compared with some other platinum-based combinations that are used to treat cervical cancer. This assumption was supported recently by results from the Japan Clinical Oncology Group (JCOG) 0505 randomised trial^9^ that showed non-inferiority for paclitaxel plus carboplatin versus paclitaxel plus cisplatin. Since VEGF concentrations in primary cervical cancer are known to affect the outcome after radiotherapy, we postulated that the addition of the potent VEGFR inhibitor cediranib to carboplatin plus paclitaxel would improve progression-free survival and response when compared with carboplatin plus paclitaxel alone.

## Methods

### Study design and participants

This phase 2 randomised, double-blind, placebo-controlled trial included patients with cervical cancer who were initially diagnosed with metastatic carcinoma or subsequently developed metastatic disease or local pelvic recurrence after radical treatment (chemoradiotherapy alone or surgery followed by chemoradiotherapy).

Eligible patients had metastatic, persistent, or locally recurrent cervical cancer that was not amenable to curative pelvic exenteration or radical radiotherapy, were at least 18 years of age, had measurable disease by Response Evaluation Criteria in Solid Tumors (RECIST) version 1.1, adequate haematological and biochemical function, ECOG performance status of 0 or 1, and a life expectancy of at least 12 weeks (appendix p 1). The main exclusion criteria were previous chemotherapy (except cisplatin given concomitantly with radiotherapy as primary treatment), previous malignancy within 5 years (except for basal cell skin cancer or in-situ breast cancer), pelvic fistulae, evidence of bowel obstruction, major surgery or substantial traumatic injury within the previous 4 weeks, non-healing wound or bone fracture, active bleeding, pre-existing thrombotic or haemorrhagic disorder, substantial proteinuria, uncontrolled hypertension, or notable allergy to carboplatin or paclitaxel.

The study was done in accordance with the Declaration of Helsinki and Good Clinical Practice guidelines; all aspects of the study received ethics approval from the National Research Ethics Service. All participants provided written informed consent before enrolment. The full study details are available in the trial protocol.

### Randomisation and masking

Eligible patients were randomly assigned in a 1:1 ratio using a minimisation algorithm incorporating a random component, stratified by disease site (local relapse only *vs* extra-pelvic metastases only *vs* local relapse and extra-pelvic metastases), disease-free survival after primary therapy or primary stage IVb disease (≤12 months *vs* >12 months *vs* treatment-naive stage IVb disease), number of lines of previous treatment (0 *vs* 1); ECOG performance status (0 *vs* 1), and investigational site (the centre or institution).

Participants were enrolled by authorised clinicians who, after obtaining patient consent, contacted the Cancer Research UK Clinical Trials Unit in Glasgow, UK, to check eligibility and request randomisation. At the end of the randomisation process, the computer randomisation system allocated every patient a unique identification number and a study drug code that was used to access masked local supply of study drug.

All patients, investigators, and trial personnel were masked to study drug allocation, with the exception of the unmasked statistician who generated and held the code break (a masked statistician did the analysis) and pharmacovigilance personnel when needed on an individual basis for safety reporting to regulatory authorities.

### Procedures

All patients received carboplatin area under the curve (AUC) of 5 (calculated by a radioisotope measurement of glomerular filtration rate) plus paclitaxel 175 mg/m^2^ (infused over 3 h) for up to six cycles repeated every 3 weeks unless progression or unacceptable toxic effects supervened. In addition to this treatment, patients were randomly assigned 1:1 to receive cediranib 20 mg orally or identical placebo orally once daily. Trial medication (cediranib or placebo) was continued after the end of cytotoxic chemotherapy until tumour progression or the development of intolerable toxic effects.

Dose modifications for haematological toxicity are in the appendix (p 2). With the exception of hypertension, short dose interruptions of cediranib study tablets were the first approach to the management of adverse events, followed by dose reduction to 15 mg if necessary. Diarrhoea was managed by loperamide (4 mg initially, then 2 mg every 2 h), oral or intravenous rehydration, and temporary cessation of cediranib medication if necessary. In any case of grade 4 thrombocytopenia, the cediranib dose was reduced to 15 mg. Hypertension was treated with a longacting calcium channel antagonist such as nifedipine (initially 5 mg three times per day, increasing to 20 mg three times per day depending on response), with temporary cessation of cediranib if necessary. The scheme for management of hypertension is shown in the appendix (p 3).

Patients were assessed for toxicity, haematology, clinical chemistry, vital signs, blood pressure, and urinalysis at the beginning of each cycle of chemotherapy and subsequently at clinic visits every 2 months. Thyroid function was tested every 2 months during follow-up. Patients were assessed for response clinically and radiologically after two, four, and six cycles of chemotherapy. Subsequently, CT or MRI scans (only one modality was used per patient) were done every 2 months until confirmed tumour progression according to RECIST version 1.1. When disease progression was confirmed, further treatment was given at the investigator's discretion. Blood was taken for plasma-soluble VEGFR2 (sVEGFR2) before treatment, at the beginning of every cycle of chemotherapy, and every 2 months after chemotherapy.

Quality of life was assessed with the European Organisation for Research and Treatment of Cancer (EORTC) QLQ-C30 and QLQ-CX24 questionnaires, which were given to patients at the onset of every cycle of chemotherapy, at the end of treatment, and every 2 months during the first year of follow-up or until confirmed progression.

### Outcomes

The primary endpoint was progression-free survival, defined as time from randomisation to progression or death (whichever occurred first). Secondary endpoints were a reduction in sVEGFR2 concentration from baseline to day 28 after initiation of chemotherapy, best response to chemotherapy (according to RECIST version 1.1 criteria), overall survival (defined as time from randomisation to death from any cause), toxic effects (assessed with National Cancer Institute Common Terminology Criteria for Adverse Events, version 4.0), and quality of life as assessed by EORTC QLQ-C30 and QLQ-CX24.

Progression and response were assessed locally by investigators, but the scan outcomes were detailed in the study case report form and subsequently checked centrally against RECIST criteria as part of the process of data cleaning before analysis.

### Statistical analysis

We calculated that we needed a sample size of 80 patients (40 per group) to record 69 progression events or deaths. The study was designed based on the methods of Rubinstein and colleagues^10^ to detect a 50% improvement in median progression-free survival with cediranib from 4 months to 6 months with 80% power, at a 20% one-sided level of significance (corresponding to a hazard ratio [HR] of 0·667). This sample size also provided 85% power at the 10% one-sided level of significance to detect a 75% increase in median progression-free survival from 4 months to 7 months (HR 0·571).

A result favouring cediranib that was significant at the one-sided 10% level would suggest that a subsequent phase 3 trial should be done. A result favouring cediranib that was significant at the one-sided 20% level but not at the one-sided 10% level would mean that other supportive data are needed—ie, a significant difference in plasma sVEGFR2 concentration from baseline to day 28 between groups (a greater reduction in the cediranib group) before a subsequent phase 3 trial could be considered. A result that was not significant at the 20% level would suggest that no further development of cediranib should be done in this setting. This method of decision-making conforms to a three-outcome approach.^11^

Data from Batchelor and colleagues' study^12^ showed that the standard deviation in the change in sVEGFR2 (log 10) concentration from baseline was 0·19 and the expected change was 0·27. Under the assumption of no change in the placebo group, to detect this difference between the groups would need 54 patients (90% power, 10% one-sided level of significance). Allowing for 10% of patients being non-assessable (ie, 10% would have disease progression or die before day 28), our study aimed to recruit 60 patients in whom this pharmacodynamic endpoint could be measured.

Efficacy analysis of progression-free and overall survival included all randomly assigned patients (intention-to-treat population). Analysis of response was restricted to those patients who, on data review, had measurable disease at baseline. Analysis of treatment delivery, quality of life, and safety was restricted to those patients who started study treatment and were analysed by original randomised group.

We estimated progression-free and overall survival using the Kaplan-Meier method. We used a Cox proportional hazards model incorporating study stratification factors to compare the study groups. We used logistic regression to compare overall response (complete or partial response) via the odds ratio between the study groups in a model incorporating study stratification factors. A 10% one-sided level of statistical significance was used for this.

We did an exploratory analysis of treatment effect heterogeneity for progression-free and overall survival by key patient or disease factors (stratification factors, age, and histology). The p value for heterogeneity was derived by comparing the likelihood ratios of two stratified Cox models: a full model with a term for treatment effect in each key factor category and a reduced model with one term for treatment effect.

We compared the change in sVEGFR2 (log 10) values from baseline to day 28 between the study groups using a *t* test. In the cediranib group, the association between this change and progression-free survival and response was assessed in the context of Cox and logistic regression models, respectively.

For the quality-of-life analysis, we imputed missing data using interpolation and last observation carried forward before deriving the AUC described by each scale. We standardised these AUC values by the time spent in the study and adjusted them by subtracting the baseline value. We compared the distributions of the adjusted standardised AUC scores between the groups with the Mann-Whitney *U* test. We adjusted the p values for the individual tests within each questionnaire for multiple comparisons using the false-discovery rate approach (calculated using the p.adjust function [fdr option] of the stats library in R version 3.1.2). With the assumption of a standard deviation of 23^13^ the study has 90% power to detect a large difference of 20^14^ at the 1% two-sided level of significance (to allow for multiple testing). A 5% level of significance was used for the quality-of-life comparisons. All p values in the text are two-sided unless stated otherwise.

SPSS version 22.0 was used for all analyses. The statistical analysis plan for the study was produced and approved before the first Data Monitoring Committee meeting. This study is registered as an International Standard Randomised Controlled Trial, number ISRCTN23516549.

### Role of the funding source

The funders had no role in study design, data collection, data analysis, data interpretation, or writing of the report. All authors had full access to all the raw data. The corresponding author had full access to all the data in the study and had final responsibility for the decision to submit for publication.

## Results

**Figure 1:**
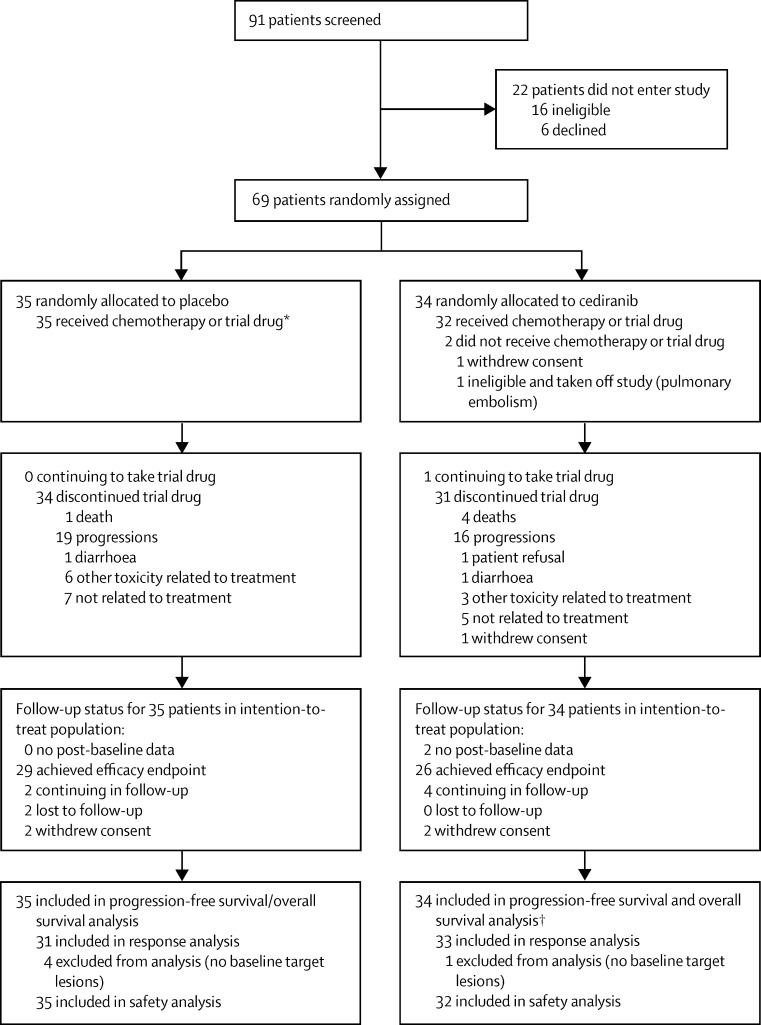
Trial profile *One patient in this group was given chemotherapy but did not receive any trial drug. †Two patients withdrew from the study before starting treatment and are censored for progression-free and overall survival at this point.

Between Aug 19, 2010, and July 27, 2012, 91 patients were screened and 69 eligible patients were enrolled from 17 cancer centres in the UK (appendix p 4). Five patients (four assigned to placebo and one assigned to cediranib) were excluded from the analysis of response because they had no measurable disease at baseline. Two patients (both in the cediranib group) were excluded from the safety analysis because they did not start chemotherapy or receive any study drug. Figure 1 shows the patient flow through the trial. Table 1 shows the baseline patient characteristics by treatment group.

The trial closed prematurely on July 27, 2012, owing to withdrawal of drug supply. Median follow-up was 24·2 months (IQR 21·9–29·5).

The administration of paclitaxel and carboplatin was similar between the treatment groups. The median total carboplatin dose delivered was an AUC of 30 (IQR 15–30) in the placebo group and AUC of 29 (25–30) in the cediranib group. The number of patients completing the full six cycles of paclitaxel and carboplatin treatment was 24 (69%) of 35 patients in the placebo group and 26 (81%) of 32 patients in the cediranib group. Seven (20%) of 35 patients in the placebo group and 11 (34%) of 32 in the cediranib group had one or more dose reduction. 23 (66%) patients in the placebo group and 19 (59%) patients in the cediranib group had one or more dose delay. The median total paclitaxel dose delivered was 1013 mg/m^2^ (IQR 699–1050) in the placebo group and 1033 mg/m^2^ (861–1051) in the cediranib group. The number of patients completing the full six cycles of treatment was 22 (63%) in the placebo group and 25 (78%) in the cediranib group. 12 (34%) patients in the placebo group had one or more dose reduction and 24 (69%) had one or more dose delays, compared with 14 (44%) and 20 (63%), respectively, in the cediranib group.

The median time on study drug was 4·4 months (IQR 1·6–5·3) in the placebo group and 4·4 months (2·5–6·7) in the cediranib group. Compliance with the study drug was good in both groups; the median percentage of doses missed was 3% (IQR 0–8) in the placebo group and 7% (1–18) in the cediranib group. Disease progression or death was the most common reason for treatment discontinuation in both groups (19 [54%] of 35 patients in the placebo group *vs* 20 [63%] of 32 patients in the cediranib group). Seven (20%) of 35 patients in the placebo group and four (13%) of 32 in the cediranib group discontinued treatment because of adverse events.

All efficacy analyses of progression-free and overall survival were done on the intention-to-treat population. Two patients in this population (both in the cediranib group) withdrew before starting study treatment, and therefore had no survival, trial drug, or chemotherapy data available. These patients were both censored for progression-free and overall survival before any events on study and therefore their data made no contribution to the comparison of the study groups, although they are included in the Kaplan-Meier plots.

**Figure 2:**
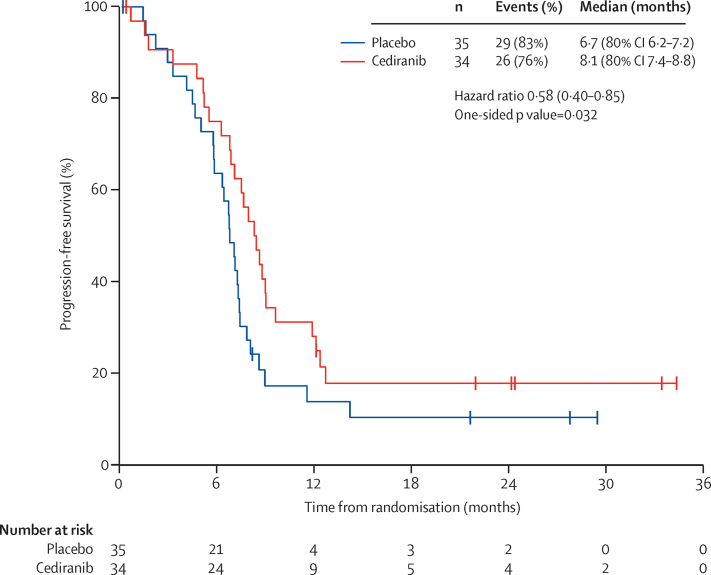
Progression-free survival

55 progression-free survival events were recorded, which means that by post-hoc calculations, the study has roughly 80% power at the 20% one-sided level of significance to detect a 60% increase in progression-free survival, or 80% power at the 10% one-sided significance level to detect a 75% increase in median progression-free survival (hazard ratio [HR] 0·625 or 0·571, respectively). The median progression-free survival of patients receiving cediranib was 8·1 months (80% CI 7·4–8·8) compared with 6·7 months (6·2–7·2) for those in the placebo group. The HR for cediranib versus placebo, adjusted for stratification factors, was 0·58 (80% CI 0·40–0·85, one-sided p=0·032; figure 2). An exploratory examination of heterogeneity in progression-free survival by stratification factors, age, and histology (appendix p 7) suggested that the effect of cediranib on progression-free survival might depend on length of disease-free survival after primary therapy (p=0·047) and disease site (p=0·062) but not age, histological subtype, number of previous treatments, or performance score at randomisation.

**Figure 3:**
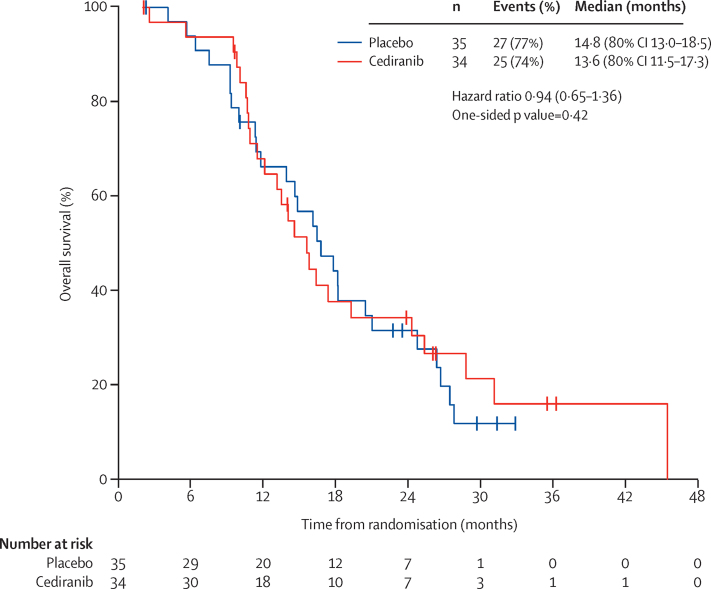
Overall survival

27 patients died in the placebo group and 25 died in the cediranib group. Median overall survival did not differ significantly between the two treatment groups (figure 3). An exploratory examination of heterogeneity suggested that the effect of cediranib on overall survival might depend on disease site (test of treatment effect heterogeneity p=0·034; patients with extra-pelvic metastatic disease only derive a greater benefit compared to those with local relapse (with or with metastatic disease), but not on age, histological subtype, length of disease free survival, number of previous treatments, or performance status score at randomisation (apppendix p 8).

The proportion of patients with an overall response in the cediranib group was 64% (three [9%] of 33 patients had a complete response and 18 [55%] had a partial response) compared with 45% in the placebo group (none of 31 patients had a complete response and 14 [45%] had a partial response). The odds ratio from logistic regression when adjusted for stratification factors for cediranib versus placebo was 2·23 (80% CI 1·06–4·69; 10% one-sided p=0·084).

**Figure 4:**
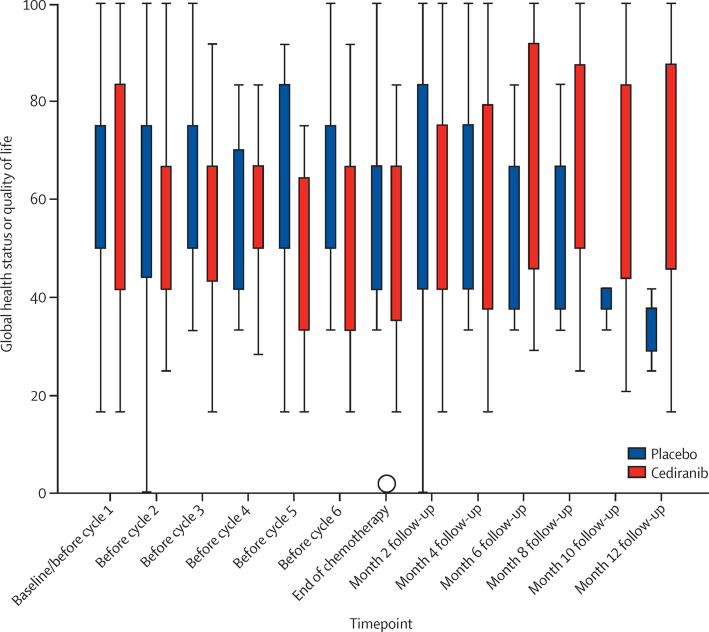
European Organisation for Research and Treatment of Cancer QLQ-C30 global health status by timepoint The circle is an outlier datapoint.

Global health status as assessed by the EORTC QLQ-C30 questionnaire is shown in figure 4 (details of questionnaire completion are in appendix p 5). AUC analysis of the data over the whole study period showed no significant difference for this scale. The median standardised adjusted AUC was −5·4 (95% CI −13·1 to −1·0) in the placebo group and −11·1 (−20·8 to −7·4) in the cediranib group (p=0·50, after adjustment for multiple comparisons). There was a significantly worse quality of life associated with diarrhoea in the cediranib group (median difference in standardised adjusted AUC 18 [95% CI 5–37], p=0·030, after adjustment for multiple comparisons). No other scales derived from the EORTC QLQ-C30 or EORTC QLQ-CX24 questionnaires had significant differences between the study groups (appendix p 5).

Table 2 shows the laboratory and non-laboratory adverse events that occurred in at least 10% of patients in either group during the chemotherapy and study drug period. Grade 3 or worse adverse events that occurred in more than 10% of patients were diarrhoea (five [16%] of 32 patients in the cediranib group *vs* one [3%] of 35 patients in the placebo group), fatigue (four [13%] *vs* two [6%]), leucopenia (five [16%] *vs* three [9%]), neutropenia (10 [31%] *vs* four [11%]), and febrile neutropenia (five [16%] *vs* none [0%]). Serious adverse events occurred in 18 patients in the placebo group (30 events) and 19 patients in the cediranib group (41 events). The remaining grade 3 or greater laboratory and non-laboratory adverse events (those associated with events occurring in <10% of patients) are listed in appendix p 6. Although there was no difference in grade 3 or 4 hypertension between the treatment groups, grade 1–2 hypertension occurred in 19 (59%) of patients in the cediranib group and eight (23%) of patients in the placebo group. The incidence of grade 2 hypertension was higher in the cediranib group (11 [34%] of 32 patients) than in the placebo group (three [9%] of 35 patients). No cases of grade 4 hypertension occurred. Five patients in the cediranib group reported a total of five occurrences of febrile neutropenia (two grade 4 and three grade 3); all were recorded as being unrelated to the study drug. During the trial drug only maintenance period, toxic effects were infrequent (appendix p 6). One patient died of a colon perforation after one cycle of carboplatin, paclitaxel, and cediranib. The role of cediranib in this death is uncertain. The tumour was adjacent to the colon before the start of therapy.

**Figure 5:**
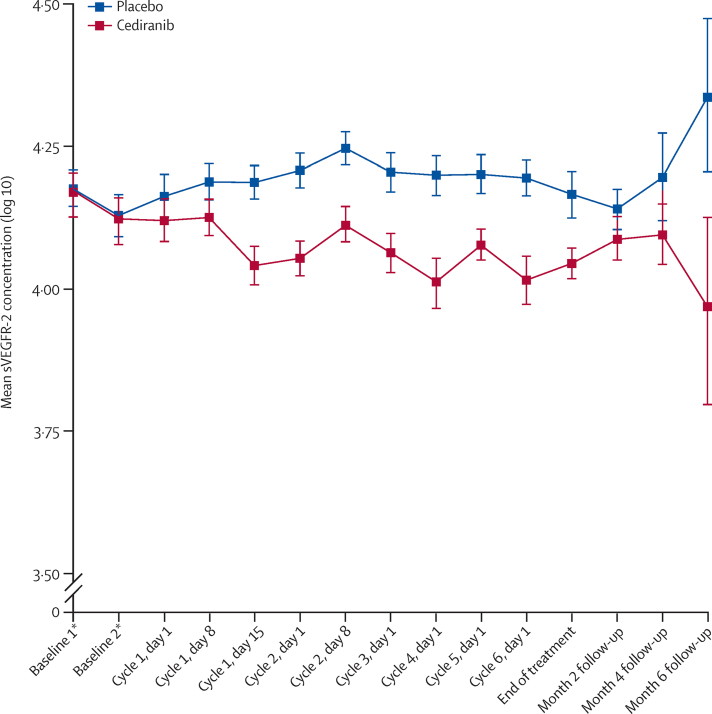
Mean sVEGFR2 concentration (log 10) by timepoint Error bars are standard errors. sVEGFR2=plasma-soluble VEGFR2. *Two baseline measurements were taken for each patient (these are replicate results).

61 (88%) of 69 patients gave consent for blood samples to be taken for sVEGFR2 marker analysis. Mean sVEGFR2 concentration increased in the placebo group and decreased in the cediranib group from baseline to day 28 (cycle 2, day 8; figure 5). The point estimate for the difference (cediranib–placebo) between the treatment groups in change in sVEGFR2 (log 10) from baseline to day 28 (cycle 2, day 8) was −0·108 (80% CI −0·147 to −0·070; 10% one-sided p=0·00077). This estimate is based on 22 patients in the placebo group and 18 patients in the cediranib group for whom data were available at both time points. Analyses showed no evidence for an association between the change in sVEGFR2 (log 10) concentration from baseline to day 28 and either progression-free survival (p=0·90, n=18) or response (p=0·22, n=17) in the cediranib group.

## Discussion

This randomised, placebo-controlled, phase 2 trial shows that the addition of cediranib to carboplatin and paclitaxel chemotherapy resulted in longer progression-free survival and a greater proportion of patients achieving an overall response than did carboplatin and paclitaxel alone in patients with metastatic or recurrent cervical cancer.

Although cross-trial comparisons should be interpreted with caution, the response and progression-free survival results in the control (placebo) group of our study were similar to other published series; indeed, the progression-free survival in our study was longer than that reported in other series. Lorusso and colleagues^15^ did a systematic review of the published literature comparing cisplatin and carboplatin plus paclitaxel-based chemotherapy for recurrent or metastatic cervical cancer, for which they included 17 eligible studies. The proportion of patients with an objective response for the carboplatin-based combination was 48·5%, with a median progression-free survival of 5 months compared with 45% of patients achieving a response and a progression-free survival of 6·7 months in this study. Survival in the control group exceeded our expectation based on our previous experience in a phase 2 trial of docetaxel and gemcitabine.^16^ However, our study might have enrolled a group of patients who had a better than expected prognosis. In comparison with JCOG0505,^9^ fewer patients were enrolled who presented with local relapse only (13% in our study *vs* 38% in JCOG0505). Similarly, in our study, a disease-free survival after primary therapy of longer than 12 months occurred in 48% of patients (compared with 23% in JCOG0505), which is a good prognostic feature. Although patients in our trial might have had a better prognosis than those reported in other studies, it should be noted that the two treatment groups were well balanced for prognostic factors.

The increase in response and progression-free survival in the cediranib group was achieved with an increase in neutropenia, febrile neutropenia, diarrhoea, and hypertension. The increase in neutropenia was associated with a small but significant increase in neutropenic sepsis.

Overall survival did not differ between the two groups but, since the study was curtailed prematurely owing to problems with drug supply at a recruitment of 69 patients, and the trial was powered for the primary endpoint of progression-free survival, the trial was not adequately powered to show a difference in overall survival. However, there are a small number of long-term survivors in both the cediranib and placebo groups (figure 2) with seven patients in each group alive at 24 months.

Although exploratory tests for treatment heterogeneity suggested that the effect of cediranib might be stronger in patients with metastatic disease (for both overall and progression-free survival) and with a disease-free interval longer than 12 months (for progression-free survival), these observations should be treated with caution in view of the modest levels of significance recorded (even before consideration of adjustments for multiple testing) and the small number of patients in each category.

Overall, our data show that mean plasma sVEGFR2 concentration decreased from baseline after the first cycle of chemotherapy in the cediranib group and increased in the placebo group. There was a significant difference in the change in sVEGFR2 concentration from baseline to 28 days between the two groups. Our study is the first trial to investigate the efficacy of cediranib in cervical cancer and this secondary endpoint was chosen to provide proof-of-principle for cediranib inhibition of VEGF signalling in the disease. In a phase 1 trial of cediranib in advanced solid tumours, time-dependent and dose-dependent reductions in sVEGFR2 concentrations were recorded.^17^ A dose of 20 mg led to a roughly 22% reduction from baseline in sVEGFR2 concentration at day 28. In a phase 1 trial of cediranib in acute myeloid leukaemia, Fiedler and colleagues^18^ also reported time-dependent reductions in in plasma sVEGFR2 levels. Batchelor and co-workers^19^ reported an average 36% reduction from baseline at day 28 in sVEGFR2 concentration in 16 patients with glioblastoma treated with a 45 mg dose of cediranib. The reductions in sVEGFR2 concentration recorded in our trial are consistent with the findings from other cancers and support its use as a biomarker of the biological activity of cediranib. Although we found no evidence for an association between a decrease in sVEGFR2 and tumour response or progression-free survival in the cediranib group, the number of evaluable patients was small.

Before we began our study, a Gynecologic Oncology Group (GOG) phase 2 study^20^ (GOG 227c) showed partial responses to bevacizumab monotherapy in 11% of patients with metastatic cervical cancer with a median response time of 6·21 months (range 2·83–8·28); all patients received bevacizumab as second-line or third-line treatment. Since the end of recruitment to our study, GOG has shown the benefit of adding bevacizumab to either cisplatin and paclitaxel or topotecan and paclitaxel given as the initial treatment for recurrent or metastatic cervical cancer. The addition of bevacizumab to chemotherapy compared with chemotherapy alone increased the response to chemotherapy (48% *vs* 36%, p=0·008), progression-free survival (8·2 months *vs* 5·9 months, HR 0·68 [95% CI 0·54–0·82]), and overall survival (17·0 months *vs* 13·3 months, HR 0·68 [98% CI 0·54–0·95).^21^

The GOG study using a monoclonal antibody to VEGF and our trial of a VEGFR tyrosine kinase inhibitor shows the value of targeting angiogenesis in combination with chemotherapy in the management of metastatic or relapsed cervical cancer. Since VEGF expression is an important factor affecting the outcome of patients treated with radiotherapy, combinations of anti-angiogenic agents and radiotherapy could be of value in locally advanced disease. Although side-effects such as diarrhoea might be increased, our experience is that diarrhoea caused by cediranib can be controlled through the use of drugs such as loperamide. No deterioration in overall quality of life occurred, which was also the case in the GOG 240 phase 3 trial of bevacizumab.^22^ Both studies suggest that patients who progress following anti-angiogenesis therapy might be suitable for investigation of novel second-line treatments. In view of the impressive overall survival advantage shown in GOG 240, the value of a direct comparison between bevacizumab and cediranib is debatable, but the evidence that both agents are effective opens up the potential for studies of multi-anti-angiogenic combinations, sequencing of anti-angiogenics, or trials of anti-angiogenics and biological agents with pre-existing evidence of synergy, including poly-ADP ribose polymerase inhibitors.^23^

Phase 3 trials have shown no benefit of the addition of cediranib to paclitaxel and carboplatin in lung cancer,^24^ to standard combination chemotherapy in colorectal cancer,^25^, ^26^ or to lomustine in glioblastoma.^19^ The absence of clinically meaningful efficacy in lung and colorectal cancer led AstraZeneca to stop its sponsored development of the drug in 2011, which led to the drug supply problems in our study.^27^ Since then, an investigator-led phase 3 trial^28^ in women with recurrent ovarian cancer showed that cediranib given concurrently with platinum-based chemotherapy improved progression-free survival and, when continued as maintenance therapy, significantly improved both progression-free and overall survival. More recently, a phase 2 trial^23^ showed that the combination of cediranib with olaparib nearly doubled progression-free survival compared with olaparib alone in women with platinum-sensitive recurrent ovarian cancer. As a result, the present focus of cediranib development in gynaecological cancers is in combination with a PARP inhibitor in patients with either platinum-sensitive or platinum-resistant ovarian cancer. Our finding supports the ongoing assessment cediranib in patients with cervical cancer.

## Figures and Tables

**Table 1:** Baseline characteristics

		**Placebo (n=35)**	**Cediranib (n=34)**
Age (years)		44 (34–53)	44 (37–60)
ECOG performance status at randomisation			
	0	14 (40%)	17 (50%)
	1	21 (60%)	17 (50%)
Treatment-naive stage IVb disease		3 (9%)	3 (9%)
Disease site			
	Local relapse only	3 (9%)	6 (18%)
	Extra-pelvic metastases only	12 (34%)	9 (26%)
	Local relapse and extra-pelvic metastases	20 (57%)	19 (56%)
Time from first pathological diagnosis to relapse, weeks*		61 (32–129)	59 (35–145)
Disease-free survival after primary therapy/primary stage IVb			
	≤12 months	16 (46%)	14 (41%)
	>12 months	16 (46%)	17 (50%)
	Stage IVb	3 (9%)	3 (9%)
Histology†			
	Squamous	26 (74%)	21 (64%)
	Adenocarcinoma	7 (20%)	7 (21%)
	Mixed	1 (3%)	4 (12%)
	Other	1 (3%)	1 (3%)
Degree of differentiation†			
	Well	5 (14%)	1 (3%)
	Moderate	12 (34%)	13 (39%)
	Poor	11 (31%)	13 (39%)
	Unknown	7 (20%)	6 (18%)
Previous treatment†			
	Previous radiotherapy	32 (91%)	30 (91%)
	Previous chemotherapy (cisplatin with radiotherapy)	29 (83%)	27 (82%)
	Previous surgery	19 (54%)	18 (55%)

Data are median (IQR) or n (%).

* For this row, n=25 in placebo group and n=28 in cediranib group.

† Data available for 33 patients in the cediranib group.

**Table 2:** Laboratory and non-laboratory adverse events occurring in at least 10% of patients in either group during chemotherapy and study drug period

	**Placebo (n=35)**			**Cediranib (n=32)**		
	Grade 1–2	Grade 3	Grade 4	Grade 1–2	Grade 3	Grade 4
Non-laboratory adverse events*						
Abdominal pain	2 (6%)	1 (3%)	0 (0%)	4 (13%)	0 (0%)	0 (0%)
Alopecia	20 (57%)	1 (3%)	0 (0%)	17 (53%)	0 (0%)	0 (0%)
Anaemia	6 (17%)	3 (9%)	0 (0%)	3 (9%)	1 (3%)	1 (3%)
Anorexia	4 (11%)	0 (0%)	0 (0%)	7 (22%)	1 (3%)	0 (0%)
Arthralgia	4 (11%)	0 (0%)	0 (0%)	3 (9%)	1 (3%)	0 (0%)
Constipation	21 (60%)	0 (0%)	0 (0%)	16 (50%)	2 (6%)	0 (0%)
Diarrhoea	14 (40%)	1 (3%)	0 (0%)	24 (75%)	5 (16%)	0 (0%)
Dysgeusia	0 (0%)	0 (0%)	0 (0%)	3 (9%)	0 (0%)	0 (0%)
Dyspnoea	0 (0%)	0 (0%)	0 (0%)	4 (13%)	0 (0%)	0 (0%)
Fatigue	27 (77%)	2 (6%)	0 (0%)	26 (81%)	4 (13%)	0 (0%)
Febrile neutropenia	0 (0%)	0 (0%)	0 (0%)	0 (0%)	3 (9%)	2 (6%)
Headache	0 (0%)	0 (0%)	0 (0%)	5 (16%)	0 (0%)	0 (0%)
Hypertension	8 (23%)	1 (3%)	0 (0%)	19 (59%)	0 (0%)	0 (0%)
Infections and infestations	3 (9%)	0 (0%)	0 (0%)	1 (3%)	1 (3%)	0 (0%)
Limb oedema	3 (9%)	0 (0%)	0 (0%)	2 (6%)	0 (0%)	0 (0%)
Maculopapular rash	3 (9%)	0 (0%)	0 (0%)	2 (6%)	0 (0%)	0 (0%)
Myalgia	8 (23%)	0 (0%)	0 (0%)	6 (19%)	0 (0%)	0 (0%)
Nausea	22 (63%)	1 (3%)	0 (0%)	20 (63%)	1 (3%)	0 (0%)
Oral mucositis	8 (23%)	0 (0%)	0 (0%)	11 (34%)	1 (3%)	0 (0%)
Pain	3 (9%)	0 (0%)	0 (0%)	5 (16%)	1 (3%)	0 (0%)
Paresthesia	4 (11%)	0 (0%)	0 (0%)	0 (0%)	0 (0%)	0 (0%)
Peripheral motor neuropathy	3 (9%)	0 (0%)	0 (0%)	6 (19%)	0 (0%)	0 (0%)
Peripheral sensory neuropathy	16 (46%)	0 (0%)	0 (0%)	16 (50%)	2 (6%)	0 (0%)
Proteinuria	8 (23%)	0 (0%)	0 (0%)	7 (22%)	2 (6%)	0 (0%)
Urinary tract infection	2 (6%)	1 (3%)	0 (0%)	2 (6%)	0 (0%)	0 (0%)
Vomiting	12 (34%)	2 (6%)	0 (0%)	11 (34%)	2 (6%)	0 (0%)
**Haematology**						
Anaemia	34 (97%)	1 (3%)	0 (0%)	28 (88%)	2 (6%)	1 (3%)
Neutropenia	15 (43%)	3 (9%)	1 (3%)	13 (41%)	9 (28%)	1 (3%)
Thrombocytopenia	3 (9%)	0 (0%)	1 (3%)	5 (16%)	0 (0%)	0 (0%)
Leucopenia	25 (71%)	3 (9%)	0 (0%)	22 (69%)	5 (16%)	0 (0%)
**Biochemistry**						
Albumin (low)	8 (23%)	2 (6%)	0 (0%)	7 (22%)	0 (0%)	0 (0%)
Alkaline phosphatase (high)	15 (43%)	0 (0%)	0 (0%)	15 (47%)	0 (0%)	0 (0%)
Alanine transaminase (high)	6 (17%)	0 (0%)	0 (0%)	12 (38%)	1 (3%)	0 (0%)
Aspartate transaminase (high)	2 (6%)	0 (0%)	0 (0%)	9 (28%)	0 (0%)	0 (0%)
Bilirubin (high)	1 (3%)	0 (0%)	0 (0%)	3 (9%)	2 (6%)	0 (0%)
Calcium (high)	3 (9%)	0 (0%)	0 (0%)	2 (6%)	0 (0%)	1 (3%)
Calcium (low)	3 (9%)	0 (0%)	0 (0%)	4 (13%)	1 (3%)	0 (0%)
Creatinine (high)	8 (23%)	0 (0%)	0 (0%)	6 (19%)	0 (0%)	0 (0%)
Glucose (high)	9 (26%)	0 (0%)	0 (0%)	15 (47%)	0 (0%)	0 (0%)
Potassium (high)	3 (9%)	0 (0%)	0 (0%)	5 (16%)	0 (0%)	1 (3%)
Potassium (low)	3 (9%)	1 (3%)	0 (0%)	6 (19%)	2 (6%)	0 (0%)
Sodium (low)	8 (23%)	2 (6%)	0 (0%)	12 (38%)	2 (6%)	0 (0%)

Data are n (%).

* Association with either chemotherapy or study drug must be at least “possible”. There was one possible treatment-related death in the cediranib group.
