# Supplementary material for: Cediranib combined with carboplatin and paclitaxel in patients with metastatic or recurrent cervical cancer (CIRCCa): a randomised, double-blind, placebo-controlled phase 2 trial
Source: Lancet Oncol. 2015 Nov;16(15):1515–24. doi: 10.1016/S1470-2045(15)00220-X (PMC4705431; doi:10.1016/S1470-2045(15)00220-X)

THE LANCET Oncology

Supplementary appendix

This appendix formed part of the original submission and has been peer reviewed. We post it as supplied by the authors.

Supplement to: Symonds RP, Gourley C, Davidson S, et al. Cediranib combined with carboplatin and paclitaxel in patients with metastatic or recurrent cervical cancer (CIRCCa): a randomised, double-blind, placebo-controlled phase 2 trial. *Lancet Oncol* 2015; published online Oct 14. http://dx.doi.org/10.1016/S1470-2045(15)00220-X.

Supplementary Appendix

Table 1S Eligibility

**Inclusion Criteria**
Over 18, life expectancy >12 weeks
ECOG performance status 0 or 1
Written informed consent
Histologically proven carcinoma of the cervix (squamous, adenocarcinoma or mixed adenosquamous)
Either
1. Persistent or relapsed inoperable disease after radical radiotherapy within the irradiated pelvis
2. Relapse after radical hysterectomy (after radical radiotherapy to the pelvis if appropriate)
3. Extra pelvic metastases
4. Stage IVb disease at diagnosis
Adequate haematological function – HB ≥10g/dl, neutrophils ≥1·5x10^9/l, platelets ≥100x10^9/l
Adequate renal function – radioisotope measurement of glomerular filtration rate ≥35mls/min
Adequate liver function – bilirubin ≤1·5xULN, ALT or AST ≤2·5 (or ≤5xULN if hepatic metastases), alkaline phosphatase ≤2.5xULN (or ≤5xULN if hepatic metastases present)
Adequate coagulation – prothrombin ratio/INR ≤1·5 or PTR/INR between 2·0 and 3·0 for patients onstable doses of anticoagulants
Partial thromboplastin time <1·2 x control
**Exclusion Criteria**
History of psychiatric disorder that would prevent informed consent and compliance
Prior chemotherapy except cisplatin administered along with radiotherapy as primary treatment
Relapse potentially treatable with exenterative surgery
History of pelvic fistula
Acute or sub-acute intestinal obstruction
History of inflammatory bowel disease
Active bleeding or non-healing wound, ulcer or fracture
Brain metastases, uncontrolled seizures, cerebro-vascular accidents/transient ischaemic attack, subarachnoid haemorrhage within 6 months
Significant proteinurea >1·5g over 24 hours
Poorly controlled hypertension or resting BP 150/100
Sensory or motor neuropathy ≥Grade 2
History of spinal cord compression
Patients who have been treated with potent inhibitors of CYP3A4 and 2C8 within 2 weeks of the first planned dose of cediranib including amiodaraone, clarithromycin, erythromycin, simvastatin, atorvastatin, lovastatin, montelukast, verapamil, ketoconazole, miconazole, indinovir and diltiazem (these drugs were not used during the trial period)

Table 2S Dose modifications of chemotherapy for haematological toxicity

**Delay in neutrophil recovery to ≥ 1·5 x 10^9/l**	**Delay in platelet recovery to ≥ 100 x 10^9/l**	**Dose modifications**
≤ 7 days	≤ 7 days	None
7-14 days	≤ 7 days	↓paclitaxel to 135mg/m^2<br>No change to carboplatin
≤ 7 days	7-14 days	No change to paclitaxel<br>↓ carboplatin by AUC of 1
7-14 days	7-14 days	↓paclitaxel to 135mg/m2<br>↓carboplatin by AUC of 1
> 14 days	> 14 days	Withdraw from protocol treatment

Table 3S Management of Hypertension

1	If blood pressure is >140/90 on 2 consecutive occasions more than 24 hours apart or an increase in diastolic blood pressure of at least 20mmHg continue cediranib/placebo at the same dosage and introduce treatment with a long acting calcium channel antagonist (CCA) such as nifedipine
2	If BP still >140/90 and has not responded after 24 hours increase dose of CCA
3	If BP still >140/90 and has not responded after a further 24 hours add in another anti-hypertensive agent.
4	If 48 hours later BP remains uncontrolled or continues to increase, temporarily hold cediranib until BP <140/90
5	Restart cediranib at same dose or as a dose reduction when BP ≤ 140/90
6	If BP remains uncontrolled or continues to increase despite dose reduction and maximal anti-hypertensive permanently stop cediranib

Table 4S: Sites and site recruitment

Site Name	Principal Investigator	Total no. patients recruited
Christie Hospital	Dr Susan Davidson	25
Royal Marsden Hospital (Fulham Rd)	Dr Susana Banerjee	6
St James's University Hospital	Dr David Jackson	6
Royal Marsden Hospital (Sutton)	Dr Susana Banerjee	4
Clatterbridge Centre for Oncology	Dr Rosemary Lord	3
Leicester Royal Infirmary	Professor R Paul Symonds	3
UCL Hospital	Dr Mary McCormack	3
Velindre Hospital	Dr Emma Hudson	3
Western General Hospital	Professor Charles Gourley	3
Beatson West of Scotland Cancer Centre	Professor Nick Reed	2
Barts and London NHS Trust	Dr Melanie Powell	2
Dorset Cancer Centre, Poole Hospital	Dr Maxine Flubacher	2
Musgrove Park Hospital	Dr Petra Jankowska	2
Wycombe Hospital	Dr Sally Trent	2
Maidstone Hospital	Dr Rema Jyothiramayi	1
Nottingham City Hospital	Dr Steve Chan	1
Stoke Mandeville Hospital	Dr Sally Trent	1

Table 5S: Quality of Life Questionnaire Completion

	Study Arm					
	Placebo			Cediranib		
	Completed	Expected	%	Completed	Expected	%
Baseline/Before Cycle 1	33	35	94.3%	33	34	97.1%
Prior to Cycle 2	26	32	81.3%	26	31	83.9%
Prior to Cycle 3	20	30	66.7%	24	30	80.0%
Prior to Cycle 4	24	27	88.9%	24	28	85.7%
Prior to Cycle 5	22	24	91.7%	20	25	80.0%
Prior to Cycle 6	19	22	86.4%	21	25	84.0%
End-of-chemotherapy	19	33	57.6%	18	28	64.3%
Month 2 follow-up	13	13	100.0%	13	18	72.2%
Month 4 follow-up	5	6	83.3%	11	11	100.0%
Month 6 follow-up	5	4	125.0%	7	8	87.5%
Month 8 follow-up	3	4	75.0%	5	4	125.0%
Month 10 follow-up	3	3	100.0%	3	4	75.0%
Month 12 follow-up	2	3	66.7%	4	4	100.0%

Table 6S: EORTC QLQ-C30 Analysis Results - comparison of standardised adjusted AUC

	Unadjusted two-sided p-value	Adjusted* two-sided p-value
**Global health status**		
Global health status	0.19	0.50
**Functional scales**		
Physical functioning	0.051	0.26
Role functioning	0.018	0.14
Emotional functioning	0.70	0.88
Cognitive functioning	0.27	0.51
Social functioning	0.12	0.47
**Symptom scales**		
Fatigue	0.82	0.88
Nausea and vomiting	0.99	0.99
Pain	0.36	0.61
Dyspnoea	0.82	0.88
Insomnia	0.21	0.50
Appetite loss	0.23	0.50
Constipation	0.83	0.88
Diarrhoea	***0.002***	***0.030***
Financial difficulties	0.71	0.88

*adjusted for multiple comparisons using the false-discovery rate approach

Table 7S: EORTC QLQ-CX24 Analysis Results - comparison of standardised adjusted AUC

	Unadjusted two-sided p-value	Adjusted* two-sided p-value
**EORTC QLQ-CX24**		
**Functional scales**		
Body image	0.60	0.83
Sexual activity	0.075	0.23
Sexual enjoyment	0.36	0.65
Sexual/vaginal functioning	0.86	0.87
**Symptom scales**		
Symptom experience	0.025	0.23
Lymphoedema	0.066	0.23
Peripheral neuropathy	0.15	0.33
Menopausal symptoms	0.87	0.87
Sexual worry	0.64	0.83

*adjusted for multiple comparisons using the false-discovery rate approach

Table 8S: Laboratory and Non-laboratory Adverse Events occurring in at least 10% of patients on either arm during study drug only period

	Placebo (n=32)								Cediranib (n=28)							
	Grade 0		Grade 1/2		Grade 3		Grade 4		Grade 0		Grade 1/2		Grade 3		Grade 4	
	n	%	n	%	n	%	n	%	n	%	n	%	n	%	n	%
**Non-laboratory***																
Diarrhoea	31	96.9%	1	3.1%	0	0.0%	0	0.0%	18	64.3%	9	32.1%	1	3.6%	0	0.0%
Fatigue	28	87.5%	3	9.4%	1	3.1%	0	0.0%	21	75.0%	7	25.0%	0	0.0%	0	0.0%
Peripheral sensory neuropathy	28	87.5%	4	12.5%	0	0.0%	0	0.0%	27	96.4%	1	3.6%	0	0.0%	0	0.0%
Vomiting	32	100%	0	0.0%	0	0.0%	0	0.0%	25	89.3%	3	10.7%	0	0.0%	0	0.0%
**Haematology**																
Anaemia	1	3.1%	31	96.9%	0	0.0%	0	0.0%	7	25.0%	20	71.4%	1	3.6%	0	0.0%
Neutropenia	7	21.9%	25	78.1%	0	0.0%	0	0.0%	11	39.3%	17	60.7%	0	0.0%	0	0.0%
Thrombocytopenia	7	21.9%	25	78.1%	0	0.0%	0	0.0%	11	39.3%	16	57.1%	1	3.6%	0	0.0%
Leucopenia	6	18.8%	26	81.3%	0	0.0%	0	0.0%	10	35.7%	18	64.3%	0	0.0%	0	0.0%
**Biochemistry**																
Alk Phos (High)	31	96.9%	1	3.1%	0	0.0%	0	0.0%	25	89.3%	3	10.7%	0	0.0%	0	0.0%
Creatinine (High)	32	100%	0	0.0%	0	0.0%	0	0.0%	25	89.3%	3	10.7%	0	0.0%	0	0.0%
Potassium (Low)	31	96.9%	1	3.1%	0	0.0%	0	0.0%	25	89.3%	3	10.7%	0	0.0%	0	0.0%

*Relationship to either chemotherapy or study drug must be at least "possible"

Table 9S: Laboratory and Non-laboratory Adverse Events occurring in less than 10% of patients, but 1 or more is grade 3/4/5

	Placebo										Cediranib									
	Grade 0		Grade 1/2		Grade 3		Grade 4		Grade 5		Grade 0		Grade 1/2		Grade 3		Grade 4		Grade 5	
	n	%	n	%	n	%	n	%	n	%	n	%	n	%	n	%	n	%	n	%
**Chemotherapy and trial drug Non-laboratory***					n=35										n=32					
Anaphylaxis	34	97.1%	1	2.9%	0	0.0%	0	0.0%	0	0.0%	31	96.9%	0	0.0%	1	3.1%	0	0.0%	0	0.0%
Bronchospasm	35	100%	0	0.0%	0	0.0%	0	0.0%	0	0.0%	31	96.9%	0	0.0%	1	3.1%	0	0.0%	0	0.0%
Colonic perforation	35	100%	0	0.0%	0	0.0%	0	0.0%	0	0.0%	31	96.9%	0	0.0%	0	0.0%	0	0.0%	1	3.1%
Dehydration	35	100%	0	0.0%	0	0.0%	0	0.0%	0	0.0%	30	93.8%	1	3.1%	1	3.1%	0	0.0%	0	0.0%
Hematuria	34	97.1%	0	0.0%	1	2.9%	0	0.0%	0	0.0%	32	100%	0	0.0%	0	0.0%	0	0.0%	0	0.0%
Hypotension	35	100%	0	0.0%	0	0.0%	0	0.0%	0	0.0%	31	96.9%	0	0.0%	1	3.1%	0	0.0%	0	0.0%
Lymph gland infection	35	100%	0	0.0%	0	0.0%	0	0.0%	0	0.0%	31	96.9%	0	0.0%	1	3.1%	0	0.0%	0	0.0%
Rectal hemorrhage	35	100%	0	0.0%	0	0.0%	0	0.0%	0	0.0%	31	96.9%	0	0.0%	1	3.1%	0	0.0%	0	0.0%
Vaginal discharge	34	97.1%	0	0.0%	1	2.9%	0	0.0%	0	0.0%	32	100%	0	0.0%	0	0.0%	0	0.0%	0	0.0%
Vaginal hemorrhage	34	97.1%	0	0.0%	1	2.9%	0	0.0%	0	0.0%	31	96.9%	1	3.1%	0	0.0%	0	0.0%	0	0.0%
Wound infection	35	100%	0	0.0%	0	0.0%	0	0.0%	0	0.0%	31	96.9%	0	0.0%	1	3.1%	0	0.0%	0	0.0%
**Trial drug only Non-laboratory***					n=32										n=28					
Thromboembolic event	32	100%	0	0.0%	0	0.0%	0	0.0%	0	0.0%	27	96.4%	0	0.0%	1	3.6%	0	0.0%	0	0.0%
**Biochemistry**																				
Bilirubin (High)	32	100%	0	0.0%	0	0.0%	0	0.0%	0	0.0%	26	92.9%	1	3.6%	1	3.6%	0	0.0%	0	0.0%
Calcium (Low)	32	100%	0	0.0%	0	0.0%	0	0.0%	0	0.0%	27	96.4%	0	0.0%	1	3.6%	0	0.0%	0	0.0%

*Relationship to either chemotherapy or study drug must be at least "possible"

Fig 1S Forest plot showing the effect of cediranib (experimental) versus placebo (control) on PFS (hazard ratio and 95% confidence intervals; p-values for test for heterogeneity of HR) stratified according to key patient/disease factors

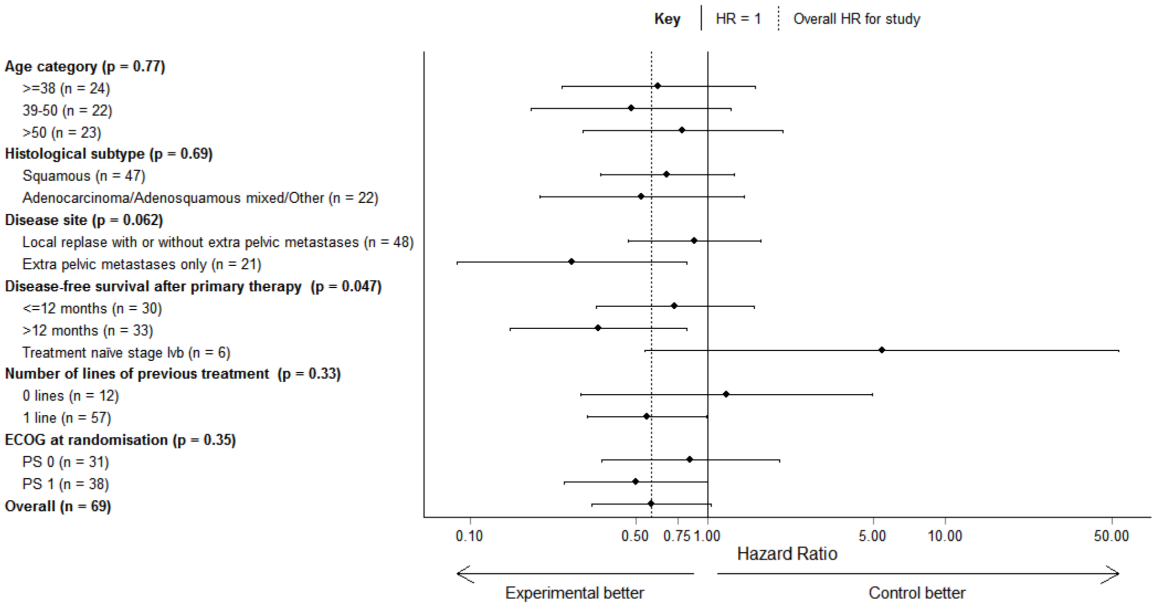

Fig 2S Forest plot showing the effect of cediranib (experimental) versus placebo (control) on OS (hazard ratio and 95% confidence intervals; p-values for test for heterogeneity of HR) stratified according to key patient/disease factors

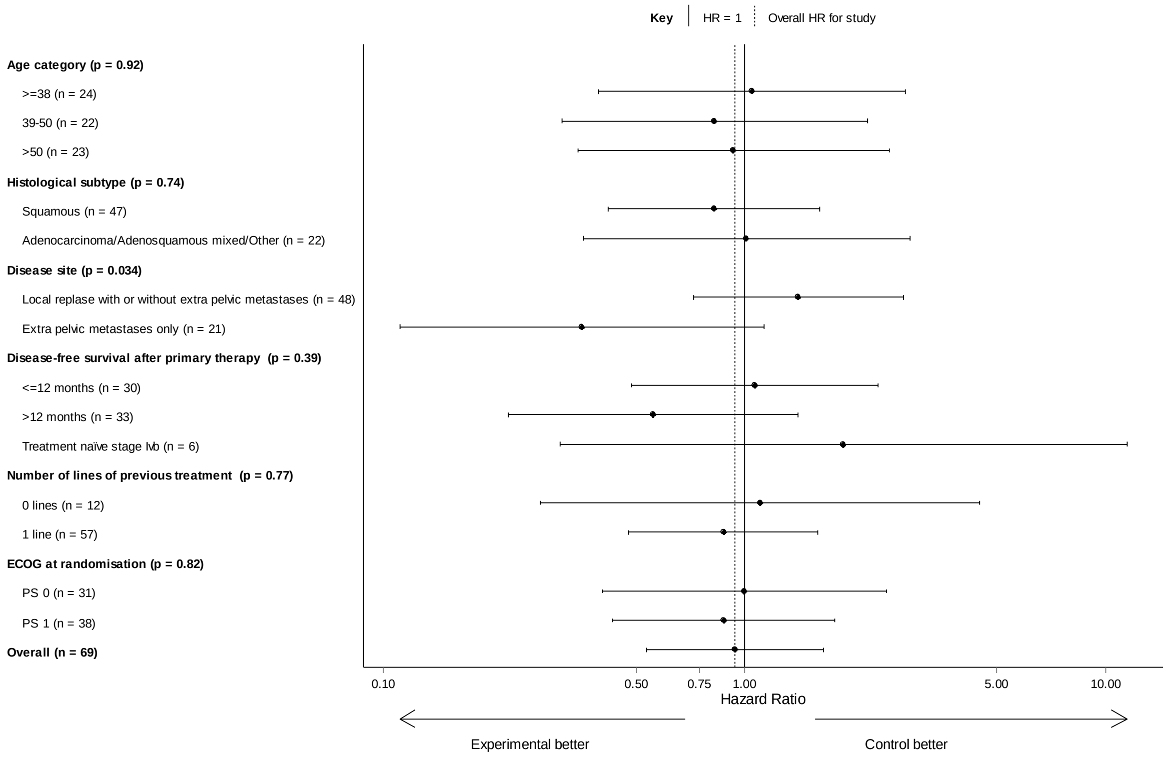

Supplement: Supplementary appendix [file mmc1.pdf]
